# Genetic testing in Poland and Ukraine: should comprehensive germline testing of *BRCA1* and *BRCA2* be recommended for women with breast and ovarian cancer?

**DOI:** 10.1017/S0016672320000075

**Published:** 2020-08-08

**Authors:** Tu Nguyen-Dumont, Pawel Karpinski, Maria M. Sasiadek, Hayane Akopyan, Jason A. Steen, Derrick Theys, Fleur Hammet, Helen Tsimiklis, Daniel J. Park, Bernard J. Pope, Ryszard Slezak, Agnieszka Stembalska, Karolina Pesz, Nataliya Kitsera, Aleksandra Siekierzynska, Melissa C. Southey, Aleksander Myszka

**Affiliations:** 1 Precision Medicine, School of Clinical Sciences at Monash Health, Monash University, Clayton, Australia; 2 Department of Clinical Pathology, The University of Melbourne, Melbourne, Australia; 3 Department of Genetics, Wroclaw Medical University, Wroclaw, Poland; 4 Institute of Hereditary Pathology of National Academy of Medical Sciences, Lviv, Ukraine; 5 Institute of Medical Sciences, University of Rzeszow, Rzeszow, Poland; 6 Melbourne Bioinformatics, The University of Melbourne, Melbourne, Australia; 7 Colorectal Oncogenomics Group, Department of Clinical Pathology, The University of Melbourne, Melbourne, Australia; 8 Department of Biotechnology and Plant Physiology, University of Rzeszow, Rzeszow, Poland; 9 Cancer Epidemiology Division, Cancer Council Victoria, Melbourne, VIC 3004, Australia

**Keywords:** *BRCA1*, *BRCA2*, breast cancer, founder mutations, genetic susceptibility, genetic testing, ovarian cancer

## Abstract

**Purpose:**

To characterize the spectrum of *BRCA1* and *BRCA2* pathogenic germline variants in women from south-west Poland and west Ukraine affected with breast or ovarian cancer. Testing in women at high risk of breast and ovarian cancer in these regions is currently mainly limited to founder mutations.

**Methods:**

Unrelated women affected with breast and/or ovarian cancer from Poland (n = 337) and Ukraine (n = 123) were screened by targeted sequencing. Excluded from targeted sequencing were 34 Polish women who had previously been identified as carrying a founder mutation in *BRCA1*. No prior testing had been conducted among the Ukrainian women. Thus, this study screened *BRCA1* and *BRCA2* in the germline DNA of 426 women in total.

**Results:**

We identified 31 and 18 women as carriers of pathogenic/likely pathogenic (P/LP) genetic variants in *BRCA1* and *BRCA2*, respectively. We observed five *BRCA1* and eight *BRCA2* P/LP variants (13/337, 3.9%) in the Polish women. Combined with the 34/337 (10.1%) founder variants identified prior to this study, the overall P/LP variant frequency in the Polish women was thus 14% (47/337). Among the Ukrainian women, 16/123 (13%) women were identified as carrying a founder mutation and 20/123 (16.3%) were found to carry non-founder P/LP variants (10 in *BRCA1* and 10 in *BRCA2*).

**Conclusions:**

These results indicate that genetic testing in women at high risk of breast and ovarian cancer in Poland and Ukraine should not be limited to founder mutations. Extended testing will enhance risk stratification and management for these women and their families.

## 1. Introduction

Women who carry a pathogenic mutation in *BRCA1* or *BRCA2* are at increased risk of developing breast and ovarian cancer. Kuchenbaecker *et al.* estimated the cumulative breast cancer risk to age 80 years to be 72% (95% confidence interval (CI) = 65–79%) and 69% (95% CI = 61–77%) for *BRCA1* and *BRCA2* pathogenic variant carriers, respectively (Kuchenbaecker *et al.*, 2017). For ovarian cancer, the cumulative cancer risk to age 80 years is estimated to be 44% (95% CI = 36–53%) and 17% (95% CI = 11–25%) for *BRCA1* and *BRCA2* pathogenic variant carriers, respectively (Kuchenbaecker *et al.*, 2017).

In many countries, genetic testing for *BRCA1* and *BRCA2* has shown clear clinical utility and validity. Evidence-based best practice guidelines are available to inform the clinical management of women who carry a *BRCA1* or *BRCA2* pathogenic variant. These guidelines support personalized risk assessment, targeted treatment regimens and informed decision-making about the use of risk-reducing medications, bilateral salpingo-oophorectomy, mammography, risk-reducing mastectomy, magnetic resonance imaging and other screening modalities.

It is well established that, as a consequence of a founder effect, different ethnic and geographical regions can have different *BRCA1* and *BRCA2* mutation spectra and prevalence rates.

Cybulski *et al.* recently reported on the mutation spectrum in *BRCA1*, *BRCA2* and other genes associated, or putatively associated, with increased risk of breast cancer in 1018 probands from multiple-case breast cancer families from Poland. In their study, three founder mutations were identified with high prevalence: *BRCA1*:c.5266dup (20%, 204/1018), *BRCA1*:c.181T>G (8.3%, 84/1018) and *BRCA1*:c.4035del (1.5%, 15/1018). Other mutations reported at lower prevalence (≤1.0%) in the 1108 familial breast cancer cases included *BRCA1*:c.3700_3704del (1.0% 10/1018), *BRCA1*:c.68_69del (0.9%, 9/1018), *BRCA1*:c.5251C>T (0.6%, 6/1018) and *BRCA1*:c.5346G>A (0.5%, 5/1108) (Cybulski *et al.*, 2019). Recurrent mutations were also reported in *BRCA2* by Cybulski *et al.*, all at a prevalence of 0.5% or below in the Polish women with familial breast cancer (Cybulski *et al.*, 2019).

The *BRCA1* mutations c.5266dupC and c.4035delA have been confirmed by haplotype analysis as founder mutations in Eastern Europe (Hamel *et al.*, 2011; Janavicius *et al.*, 2013). *BRCA1*:c.181T>G mutation carriers of Polish and Jewish ancestry have been shown to present the same haplotype (Kaufman *et al.*, 2009). Haplotype analysis of *BRCA1*:c.68_69delAG showed a common haplotype among Ashkenazi Jews (Laitman *et al.*, 2013). Currently, genetic testing in the Polish population mainly relies on testing of the founder mutations. Comprehensive *BRCA1* and *BRCA2* genetic testing could, however, identify more women with pathogenic variants, thus leading to improved cancer prevention for more women at high risk of breast and ovarian cancer.

Participants in this study were unrelated women affected with breast or ovarian cancer from south-west Poland and west Ukraine. The Polish participants had previously been genotyped for *BRCA1*:c.5266dup, *BRCA1*:c.181T>G, *BRCA1*:c.4035del, *BRCA1*:c.68_69del and *BRCA2*:c.5946del. Thirty-four women identified as carrying one of these mutations were excluded from further testing in this study. Participants recruited in Ukraine had not undergone prior genetic testing and have thus all been included in the genetic testing reported in this study.

## 2. Materials and methods

### 2.1. Study participants

The women participating in this study were unrelated women diagnosed with breast and/or ovarian cancer recruited after or during oncological treatment from Wroclaw Medical University, Lower Silesia, Poland, between 2004 and 2008, or Lviv State Oncology Regional Treatment and Diagnostic Center, Lviv, Ukraine, between 2004 and 2010, as described previously (Myszka *et al.*, 2018). The Polish cohort consisted of 238 women affected with breast cancer, 95 women affected with ovarian cancer and 4 women affected with breast and ovarian cancer. Of the 242 women with breast cancer, 95 had hereditary breast cancer, 18 had familial breast cancer and 125 were sporadic cases, according to the criteria described by Berliner *et al.* (2007). Of the 95 Polish women with ovarian cancer, 28 had hereditary ovarian cancer, 10 had familial ovarian cancer and 57 were sporadic ovarian cancer cases. All four women with breast and ovarian cancers met the criteria for hereditary disease. The Polish cohort thus consisted of 337 women, all of whom had previously been genotyped for four mutations in *BRCA1* (c.5266dup, c.181T>G, c.4035del, c.68_69del) (Table 1) and one mutation in *BRCA2* (c.5946delT). Thirty-four women were identified as carriers of one of these *BRCA1* founder mutations and were thus not included in the targeted sequence screening described in this study. No *BRCA2* c.5946delT carrier was observed.

**Table 1.** Reported prevalence of *BRCA1* founder mutations in the Polish population.

HGVS_cDNA*^a^*	HGVS_p	BIC*^b^*	MAF ExAC*^c^*	MAF Polish population	References
c.5266dup	p.(Gln1756ProfsTer74)	5382insC	0.00016	1.7 × 10^−3^ to 3.5 × 10^−3^	Gorski *et al.* (2005); Brozek *et al.* (2011)
c.181T>G	p.(Cys61Gly)	300T>G	5.64e–05	0.5 × 10^−3^ to 0.8 × 10^−3^	Gorski *et al.* (2005); Brozek *et al.* (2011)
c.4035del	p.(Glu1346LysfsTer20)	4153delA	3.68e–05	0.2 × 10^−3^	Gorski *et al.* (2005)
c.68_69del	p.(Glu23ValfsTer17)	185delAG	0.000406	<0.1 × 10^−3^	Brozek *et al.* (2011)

*^a^* Variant nomenclature according to the Human Genome Variation Society (HGVS). Transcript sequences is *BRCA1*: NM_007294.3.

*^b^* Variant nomenclature according to the Breast Cancer Information Core (BIC) (https://research.nhgri.nih.gov/bic).

*^c^* Minor allele frequency (MAF) in the ExAC database, in the non-Finnish European population minus The Cancer Genome Atlas (Lek *et al.*, 2016).

The Ukrainian cohort consisted of 112 women with breast cancer, 10 women with ovarian cancer and 1 woman with breast and ovarian cancer. Seventy-three women affected with breast cancer met the hereditary cancer criteria and 38 women met the familial cancer criteria. For one Ukrainian participant with breast cancer, insufficient information was available to classify her cancer. Of the women with ovarian cancer, six had hereditary ovarian cancer and four had familial ovarian cancer. The woman with breast and ovarian cancer met the hereditary cancer criteria. There had been no previous testing for mutations in *BRCA1* and *BRCA2* conducted in the Ukrainian participants.

All participants provided informed consent for participation in this research programme, which was approved by the Commission of Bioethics of the Institute of Hereditary Pathology of the National Academy of Medical Sciences of Ukraine, the Ethics Committee of Wroclaw Medical University (Poland), the Ethics Committee of the University of Rzeszow (Poland) and the University of Melbourne Human Research Ethics Committee (Australia).

### 2.2. Mutation screening

Amplicon-based massively parallel sequencing of the protein-coding regions and proximal intron–exon junctions of *BRCA1* (NM_007294.3) and *BRCA2* (NM_000059.3) was performed using lymphocyte-derived germline DNA and the Hi-Plex protocol (Nguyen-Dumont *et al.*, 2015). All oligonucleotides were synthesized by Integrated DNA Technologies (Coralville, IA, USA). Gene-specific primers and adapter primers were purified to standard desalting and high-performance liquid chromatography grade, respectively. All oligonucleotide sequences are available upon request. Massively parallel sequencing (150 bp paired-end) was performed on the MiSeq system (Illumina, San Diego, CA, USA). Mapping to the human reference build GRCh37 was performed using *bwa-mem* 0.7.17 (Li & Durbin, 2009). Variant calling was performed using the Java version of *VarDict* in single-sample, amplicon mode (Lai *et al.*, 2016).

### 2.3. Annotation and classification of variants

Variants were annotated using *VEP* (v.90) and loaded into *GEMINI*, according to the authors’ recommendations (Paila *et al.*, 2013; McLaren *et al.*, 2016). Classification of genetic variants in *BRCA1* and *BRCA2* was then performed in accordance with the Evidence-based Network for the Interpretation of Germline Mutant Alleles (ENIGMA) consortium's recommendations (Spurdle *et al.*, 2012). Pathogenicity calls were retrieved from the BRCA Exchange portal (http://brcaexchange.org; accessed 17 June 2019). When no information was available (i.e., variants reported as ‘no data’, ‘not available’ or ‘not yet reviewed’), variants were classified in accordance with the ENIGMA criteria. Specifically, truncating variants (i.e., nonsense, frameshift insertions or deletions and variants occurring in consensus splice junctions) were classified as pathogenic/likely pathogenic (P/LP). Missense substitutions that had not been reviewed by ENIGMA were classified as variants of unknown significance (VUS).

## 3. Results

Targeted sequencing identified a total of 31 and 18 women as carriers of a P/LP variant in *BRCA1* and *BRCA2*, respectively (Tables 2 & 3). The clinical characteristics of these women are available in Supplementary Table S1.

**Table 2.** Pathogenic and likely pathogenic*^a^ BRCA1* and *BRCA2* mutations carriers identified in 460 women affected with breast or ovarian cancer in south-west Poland and west Ukraine.

Gene	Variant type	HGVS_c*^b^*	HGVS_p*^b^*	Classification*^c^*	Number of carriers				
					Total	Pol*^d^*	Ukr*^d^*	BC*^e^*	OC*^e^*
*BRCA1^f^*	Nonsense	c.5251C>T	p.Arg1751Ter	Pathogenic	1	1	0	1	0
		c.5346G>A	p.Trp1782Ter	Pathogenic	1	1	0	1	0
	Frameshift	c.68_69del*^g^*	p.Glu23ValfsTer17	Pathogenic	3	1	2	2	1
		c.374dup	p.Gln126ProfsTer16	No data	1	1	0	0	1
		c.843_846del	p.Ser282TyrfsTer15	Pathogenic	1	1	0	1	0
		c.844_850dup	p.Gln284LeufsTer5	Pathogenic	1	0	1	0	1
		c.1510del	p.Arg504ValfsTer28	Pathogenic	1	0	1	1	0
		c.1612_1616del	p.Gln538GlyfsTer11	Pathogenic	1	1	0	0	1
		c.2217dup*^h^*	p.Val740SerfsTer3	Pathogenic	1	0	1	1	0
		c.2291_2292del	p.Val764GlyfsTer3	No data	1	0	1	1	0
		c.4035delA*^g^*	p.Glu1346LysfsTer20	Pathogenic	2	2	0	0	2
		c.5030_5033del	p.Thr1677IlefsTer2	Pathogenic	3	0	3	2	1
		c.5177_5180del	p.Arg1726LysfsTer3	Pathogenic	2	0	2	2	0
		c.5266dup*^g^*	p.Gln1756ProfsTer74	Pathogenic	35	24	11	25	12
	Splice donor	c.4357+1G>C	–	No data	1	0	1	1	0
	Missense	c.181T>G*^g^*	p.Cys61Gly	Pathogenic	10	7	3	4	6
*BRCA2^f^*	Nonsense	c.3075_3076delinsTT	p.Lys1025_Lys1026delinsAsnTer	Pathogenic	3	3	0	0	3
		c.5857G>T	p.Glu1953Ter	Pathogenic	1	1	0	1	0
		c.7721G>A	p.Trp2574Ter	Pathogenic	1	0	1	1	0
		c.8623G>T	p.Glu2875Ter	No data	1	1	0	1	0
	Frameshift	c.2945del	p.Ile982AsnfsTer9	Pathogenic	1	0	1	1	0
		c.5205_5208del	p.Gln1736IlefsTer4	Pathogenic	1	1	0	1	0
		c.6315_6318del	p.Pro2107ValfsTer11	No data	1	1	0	1	0
		c.6405_6409del	p.Asn2135LysfsTer3	Pathogenic	1	0	1	1	0
		c.6408_6414del	p.Asn2137LysfsTer29	Pathogenic	1	0	1	1	0
		c.7069_7070del	p.Leu2357ValfsTer2	Pathogenic	1	0	1	1	0
		c.9097dup	p.Thr3033AsnfsTer11	Pathogenic	1	0	1	1	0
		c.9253dup	p.Thr3085AsnfsTer26	Pathogenic	1	1	0	1	0
		c.10095delinsGAATTATATCT	p.Ser3366AsnfsTer4	Not yet reviewed	1	0	1	1	0
	Splice donor	c.475+1G>T	–	No data	3	0	3	3	0

*^a^* Genetic variants in *BRCA1* and *BRCA2* that are classified as pathogenic by the expert panel Evidence-based Network for the Interpretation of Germline Mutant Alleles (ENIGMA) (Spurdle *et al.*, 2012) as reported on the BRCA Exchange portal (http://brcaexchange.org), unreported truncating variants and variants occurring in consensus splice sites.

*^b^* Variant nomenclature based on +1 as A of ATG start codon, according to the Human Genome Variation Society (HGVS), HGVS_c for coding DNA and HGVS_p for protein variants.

*^c^* Classification according to the ENIGMA expert panel (Spurdle *et al.*, 2012), available from the BRCA Exchange portal (http://brcaexchange.org).

*^d^* Pol = Polish; Ukr = Ukrainian.

*^e^* BC = breast cancer; OC = ovarian cancer. Some women were diagnosed with both BC and OC.

*^f^* Transcript sequences are *BRCA1*: NM_007294.3 and *BRCA2*: NM_00059.3.

*^g^* Founder mutation – identified via targeted sequencing (this study) or via Sanger sequencing (prior testing) (Table 1).

*^h^* No DNA was available for validation by Sanger sequencing.

**Table 3.** Clinical characteristics of pathogenic and likely pathogenic*^a^ BRCA1* and *BRCA2* variant carriers, identified from 460 women affected with breast or ovarian cancer in south-west Poland and west Ukraine.

Cohort	Cancer type	Cancer classification*^b^*	Studied cases	Carriers of a founder mutation*^c^*	Carriers of a non-founder P/LP variant*^a^*	Carriers of any P/LP variant*^a^*
Polish	Breast	Hereditary	95	11 (11.6%)	4 (4.2%)	15 (15.8%)
		Familial	18	1 (5.5%)	1 (5.5%)	2 (11.1%)
		Sporadic	125	1 (0.8%)	2 (1.6%)	3 (2.4%)
		Total	238	13 (5.5%)	7 (2.5%)	20 (8.4%)
	Ovarian	Hereditary	28	17 (60.7%)	2 (7.1%)	19 (67.8%)
		Familial	10	0	3 (30%)	3 (30%)
		Sporadic	57	1 (1.7%)	0	1 (1.7%)
		Total	95	18 (18.9%)	5 (5.3%)	23 (24.2%)
	Breast and ovarian	Hereditary	4	3 (75%)	1 (25%)	4 (100%)
	**All Polish cases**		**337**	**34 (10.1%)**	**13** (**3.9%)**	**47** (**13.9%)**
Ukrainian	Breast	Hereditary	73	12 (16.4%)	14 (19.2%)	26 (35.1%)
		Familial	38	2 (5.3%)	4 (10.5%)	6 (15.8%)
		Sporadic	0	–	–	–
		Unknown	1	0	1	1
		Total	112	14 (12.5%)	19 (15.4%)	33 (29.5%)
	Ovarian	Hereditary	6	1 (16.7%)	1 (16.7%)	2 (33.3%)
		Familial	4	0	0	0
		Sporadic	0	–	–	–
		Total	10	1 (0.9%)	1 (0.9%)	2 (18.2%)
	Breast and ovarian	Hereditary	1	1	0	1 (100%)
	**All Ukrainian cases**		**123**	**16 (13%)**	**20** (**16.3%)**	**36** (**29.3%)**
Polish and Ukrainian	Breast	Hereditary	168	23 (13.7%)	18 (10.7%)	41 (24.4%)
		Familial	56	3 (5.4%)	5 (8.9%)	8 (14.3%)
		Sporadic	125	1 (0.8%)	2 (1.6%)	3 (2.4%)
		Unknown	1	0	1	1
		Total	350	27 (7.7%)	26 (7.4%)	53 (15.1%)
	Ovarian	Hereditary	34	18 (52.9%)	3 (8.8%)	21 (61.8%)
		Familial	14	0	3 (21.4%)	3 (21.4%)
		Sporadic	57	1 (1.8%)	0	1 (1.8%)
		Total	105	19 (18.1%)	6 (5.7%)	25 (23.8%)
	Breast and ovarian	Hereditary	5	4 (80%)	1 (20%)	5 (100%)
	**Total cases**		**460**	**50 (10.9%)**	**33** (**7.2%)**	**83** (**18.0%)**

*^a^* Genetic variants in *BRCA1* and *BRCA2* that are classified as pathogenic/likely pathogenic (P/LP) by the expert panel Evidence-based Network for the Interpretation of Germline Mutant Alleles (ENIGMA) (Spurdle *et al.*, 2012) as reported on the BRCA Exchange portal (http://brcaexchange.org), unreported truncating variants and variants occurring in consensus splice sites.

*^b^* Classification as per Berliner *et al.* (2007).

*^c^* Founder mutations in Table 1.

The 49 P/LP variants with a variant allele fraction >0.2 and total depth ≥10× were verified by Sanger sequencing, except for the case that was identified as carrying the *BRCA1* variant NM_007294.3:c.4357+1G>C, as there was insufficient DNA available. That position was covered by 1302 reads, and the variant allele fraction was 0.53 and thus highly unlikely to be a sequencing artefact.

The Polish women participating in this study were previously genotyped for mutations in *BRCA1* and *BRCA2*. The prevalence of *BRCA1* founder mutations (c.5266dup, c.181T>G, c.4035del, c.68_69del) identified prior to this study was 10.1% (34/337) (Tables 2 & 3). Targeted sequencing did not identify any additional carrier of the mutations previously tested. We detected five carriers of a non-founder P/LP *BRCA1* variant and eight carriers of a P/LP *BRCA2* variant (3.9% combined prevalence of non-founder mutations) (Table 3).

Among the Ukrainian women, the prevalence of founder mutations was 13% (16/123) (Table 3). We identified 11 carriers of *BRCA1*:c.5266dup, 3 carriers of *BRCA1*:c.181T>G and 2 carriers of *BRCA1*:c.68_69del. We did not observe *BRCA1*:c.4035del in the Ukrainian cohort. There were 10/123 carriers of a P/LP variant in *BRCA1* and 10/123 carriers of a P/LP variant in *BRCA2* (16.3% combined prevalence of non-founder P/LP variants) (Table 3). *BRCA1*:c.5030_5033del was observed in three unrelated women with breast cancer from Ukraine, all of whom having a family history of cancer (one had a sister with breast cancer and two had mothers with ovarian cancer) (Supplementary Table S1).

Targeted sequencing also identified 11 and 27 rare missense substitutions in *BRCA1* and *BRCA2*, respectively, which are currently classified as VUS (Table 4).

**Table 4.** Variants of unknown significance*^a^* in *BRCA1* and *BRCA2* identified by Hi-Plex targeted sequencing, in 426 women affected with breast or ovarian cancer in south-west Poland and west Ukraine.

Gene	HGVS_c*^b^*	HGVS_p*^b^*	MAF ExAC*^c^*	CADD*^d^* PHRED score	REVEL*^d^* score	Number of carriers			
						Pol*^e^*	Ukr*^e^*	BC*^f^*	OC*^f^*
*BRCA1^g^*	c.5047G>C	p.Glu1683Gln		23.5	0.525	1	0	0	1
	c.5005G>T	p.Ala1669Ser	5.53E–05	25.5	0.704	1	0	1	0
	c.4730C>A	p.Ser1577Tyr	1.85E–05	3.349	0.574	1	0	1	0
	c.4036G>A	p.Glu1346Lys	7.36E–05	25.3	0.546	0	1	1	0
	c.3092T>G	p.Ile1031Ser		10.5	0.551	1	0	0	1
	c.2686A>T	p.Ser896Cys		22.4	0.44	1	0	0	1
	c.1441C>G	p.Leu481Val		18.7	0.668	2	1	0	3
	c.429A>C	p.Glu143Asp	1.84E–05	22.5	0.57	1	0	1	0
	c.358G>A	p.Asp120Asn		23.4	0.384	1	0	1	0
	c.116G>A	p.Cys39Tyr		37	0.932	1	0	1	0
*BRCA2^g^*	c.353G>A	p.Arg118His	9.59E–05	9.169	0.334	2	0	2	0
	c.955A>G	p.Asn319Asp		2.463	0.216	1	0	1	0
	c.1040A>C	p.Gln347Pro		7.396	0.203	0	1	1	0
	c.1292C>T	p.Thr431Ile		0.115	0.26	1	0	1	0
	c.1514T>C	p.Ile505Thr	0.00108	5.755	0.216	1	0	1	0
	c.1556G>C	p.Ser519Thr		1.52	0.244	1	0	1	0
	c.1645A>G	p.Lys549Glu		4.923	0.133	1	0	1	0
	c.1792A>G	p.Thr598Ala	0.00371	6.88	0.236	0	1	1	0
	c.2153A>C	p.Glu718Ala		8.074	0.219	0	1	1	0
	c.2803G>A	p.Asp935Asn	0.000832	1.58	0.092	3	1	4	0
	c.3515C>G	p.Ser1172Trp		13.93	0.195	1	0	0	1
	c.4696A>G	p.Thr1566Ala		0.683	0.229	1	0	0	1
	c.5479A>G	p.Ile1827Val		0.356	0.175	1	0	1	0
	c.5737T>C	p.Cys1913Arg	1.84E–05	0.993	0.287	1	0	1	0
	c.6317T>C	p.Leu2106Pro	0.00013	15.92	0.076	1	0	1	0
	c.6455C>A	p.Ser2152Tyr	0.000468	14.74	0.512	1	0	0	1
	c.7994A>G	p.Asp2665Gly	0.000168	32	0.801	1	0	1	0
	c.8182G>A	p.Val2728Ile	0.00326	1.787	0.462	2	1	2	1
	c.9038C>T	p.Thr3013Ile	0.000353	10.91	0.368	0	1	1	0
	c.9371A>T	p.Asn3124Ile	1.84E–05	28.2	0.828	0	1	0	1

*^a^* Missense substitutions in *BRCA1* and *BRCA2* that are present at less than 1% in ExAC, that have not been reviewed yet or are classified as variants of unknown significance on BRCA Exchange by the Evidence-based Network for the Interpretation of Germline Mutant Alleles (ENIGMA) expert panel (Spurdle *et al.*, 2012).

*^b^* Variant nomenclature based on +1 as A of ATG start codon, according to the Human Genome Variation Society (HGVS), HGVS_c for coding DNA and HGVS_p for protein variants.

*^c^* ExAC non-Finnish European population minus The Cancer Genome Atlas (Lek *et al.*, 2016).

*^d^* CADD (Kircher *et al.*, 2014); REVEL (Ioannidis *et al.*, 2016).

*^e^* Pol = Polish; Ukr = Ukrainian.

*^f^* BC = breast cancer; OC = ovarian cancer.

*^g^* Transcript sequences are *BRCA1*: NM_007294.3 and *BRCA2*: NM_00059.3.

## 4. Discussion

The overall prevalence of P/LP *BRCA1* and *BRCA2* variants in the Polish women in this study was 13.9% (47/337) (Table 3). Of these, over a quarter were non-founder mutations (27.7%, 13/47). Our findings are consistent with a recent report by Kowalik *et al.*, who screened for *BRCA1* and *BRCA2* in Polish women who qualified for genetic testing and identified 161 P/LP variants, 64% (103/161) of which were founder mutations and 24.8% (40/161) of which were non-founder P/LP variants (Kowalik *et al.*, 2018).

To the best of our knowledge, our study is the first to report the mutation screening of the complete coding regions of *BRCA1* and *BRCA2* in Ukrainian women affected with breast and/or ovarian cancer. In the Ukrainian women, the overall prevalence of P/LP *BRCA1* and *BRCA2* variants was 29.3% (36/123) (Table 3). There was no difference in the proportion of founder and non-founder mutations (44.4%, 16/36, and 55.5%, 20/36, respectively).

The higher overall prevalence of P/LP variants observed in the Ukrainian participants (29.3% versus 13.9% in the Polish participants) is likely due to differences in selection criteria. Among the Ukrainian women, 99% (122/123) of participants met the criteria for hereditary (65%, 80/123) or familial cancer (34%, 42/123), whereas the Polish cohort included a majority of sporadic cancers (54%, 182/337) (Berliner *et al.*, 2007). Hereditary and familial cancers in the Polish cohort accounted for only 38% (127/337) and 8% (28/337) of all participants, respectively (Table 3).

Overall, non-founder variants represented 43.9% (18/41), 62.5% (5/8) and 66.7% (2/3) of all P/LP variants observed in women affected with hereditary, familial and sporadic breast cancer, respectively (Table 3). In women affected with ovarian cancer, non-founder variants represented 14.3% (3/21) and 100% (3/3) of all P/LP variants observed in hereditary and familial cancer, respectively. Expanding genetic testing beyond genotyping for founder variants has thus enabled us to identify 33 women carrying a clinically actionable variant who will be able to receive personalized clinical advice for themselves and their family. These results support the utility of comprehensive gene testing of *BRCA1* and *BRCA2* in Polish and Ukrainian patients, especially in women with hereditary and familial cancers.

In addition to P/LP variants, our study identified 38/427 carriers (7.7%) of rare missense variants of unknown clinical significance. Missense substitutions may result in variant proteins with functions ranging from normal to severely altered. Therefore, this group of variants is highly likely to be made up of variants with differing levels of associated risks (including none). There are substantial ongoing efforts by ENIGMA to classify VUS in *BRCA1* and *BRCA2* (Vallee *et al.*, 2012). However, methods such as calibrated functional assays are essential for enabling variant classification, and we currently lack the evidence base from which to interpret and report most missense substitutions.

Our study applied an amplicon-based targeted sequencing methodology that is not designed to detect copy number variations (CNVs). The inherent nature of targeted sequencing poses substantial challenges for the detection of these variants. A number of software tools for CNV detection from targeted sequencing data have recently emerged to try to address this gap (Li *et al.*, 2012; Ellingford *et al.*, 2017; Kerkhof *et al.*, 2017). However, they are developed for probe-based enrichment rather than amplicon-based methodologies and, to date, multiplex ligation-dependent probe amplification remains the gold standard method for the clinical identification of such events.

## 5. Conclusions

Our results show that performing comprehensive genetic testing of *BRCA1* and *BRCA2* instead of testing for founder mutations only will be highly valuable in Poland and Ukraine. Massively parallel sequencing is an effective way of performing comprehensive genetic testing of *BRCA1* and *BRCA2* that will increase the detection rate of clinically actionable variants and thus enhance risk assessment and management for these women and their families.

## Supplementary material

For supplementary material accompanying this paper visit http://dx.doi.org/10.1017/S0016672320000075. click here to view supplementary material

## Availability of data and material

The datasets used and/or analysed during the current study are available from the corresponding author on reasonable request.
