# Supplementary material for: Genetic testing in Poland and Ukraine: should comprehensive germline testing of BRCA1 and BRCA2 be recommended for women with breast and ovarian cancer?
Source: Genet Res (Camb). 2020 Aug 8;102:e6. doi: 10.1017/S0016672320000075 (PMC7443769; doi:10.1017/S0016672320000075)
Supplement: Supplementary file 1 [file S0016672320000075sup001.pdf]

Cohort	Screening method	Gene	HGVS_c	Cancer type	Age at diagnosis	Histology	Cancer classification	No of breast cancers in the family	No of ovarian cancers in the family	Family history
POL	NGS	BRCA1	NM_007294.3:c.1612_1616del	ovarian	48	serous	Familial	1		breast cancer in niece dx at 36 died at 41
POL	NGS	BRCA1	NM_007294.3:c.374dup	ovarian	49	serous	Hereditary		3	ovarian cancer in mother dx at 37 died at 40, ovarian cancer in grandmother and her sister (maternal side), ovarian cancer in aunt's daughter dx at 47
POL	NGS	BRCA1	NM_007294.3:c.5251C>T	breast	47	ductal	Hereditary	1		breast cancer in mother dx at 50 died at 52; stomach cancer in mother's brother died at 50; cancer stomach in father's sister died at 30; larynx cancer in father's brother died at 70; stomach cancer in father's niece died at 50
POL	NGS	BRCA1	NM_007294.3:c.5266dup	ovarian	56	adenocarcinoma	Sporadic			colorectal cancer in father's brother, stomach cancer in second father's brother, lang cancer in son, uterine cancer and melanoma in mother dx at 72 died 78, stomach cancer in grandmother (mother side) died at 56
POL	NGS	BRCA1	NM_007294.3:c.5346G>A	breast	58	medullary	Hereditary	2	1	breast cancer in mother dx at 41; ovarian cancer in mother's sister died at 50; stomach cancer in mother's brother died at 50; colorectal cancer in second mother's brother; breast and ovarian cancers in sister dx at 40 died at 48
POL	NGS	BRCA1	NM_007294.3:c.843_846del	breast	42	ductal	Familial	1		beast cancer in sister
POL	PCR	BRCA1	NM_007294.3:c.181T>G	ovarian bilateral	38	endometroid	Hereditary			sarcoma in mother dx at 40
POL	PCR	BRCA1	NM_007294.3:c.181T>G	ovarian bilateral	40	serous	Hereditary			stomach cancer in mother dx at 59 and in her three sisters
POL	PCR	BRCA1	NM_007294.3:c.181T>G	ovarian	53	serous	Hereditary		2	ovarian cancer in mother dx at 61died at 65; ovarian cancer in mother's sister died at 60; bone cancer in second mother's sister died at 70
POL	PCR	BRCA1	NM_007294.3:c.181T>G	ovarian	57	NA	Hereditary		1	ovarian cancer in mother dx at 68 died 69
POL	PCR	BRCA1	NM_007294.3:c.181T>G	ovarian bilateral	54	endometroid	Hereditary			pancreas cancer in mother died at 56 and grandmother (mother side) died 60
POL	PCR	BRCA1	NM_007294.3:c.181T>G	ovarian bilateral	48	endometroid	Hereditary			uterine cancer in mother died at 73; lung cancer in mother's sister died at 70
POL	PCR	BRCA1	NM_007294.3:c.181T>G	breast	37	NA	Hereditary	2		breast cancer in mother dx at 63 and sister dx at 45, uterine cancer in the second sister
POL	PCR	BRCA1	NM_007294.3:c.4035delA	ovarian bilateral	58	serous	Hereditary		1	ovarian cancer in sister died at 54; lung cancer in brother died at 60
POL	PCR	BRCA1	NM_007294.3:c.4035delA	ovarian	52	NA	Hereditary		2	ovarian cancer in mother dx at 62 died 89; ovarian cancer in sister dx at 52 died 55
POL	PCR	BRCA1	NM_007294.3:c.5266dup	breast	41	ductal	Hereditary		2	ovarian cancer in sister dx at 39; ovarian cancer in mother dx at 35 died at 40; colorectal cancer in father died at 73
POL	PCR	BRCA1	NM_007294.3:c.5266dup	breast	40	ductal	Hereditary	1		breast cancer in mother dx at 41died at 42; lung cancer in two mother's brothers died at > 50; bilateral breast cancer in daughter of one of this mother's brothers, lung cancer in grandfather (maternal side)
POL	PCR	BRCA1	NM_007294.3:c.5266dup	breast	36	medullary	Hereditary	2		breast cancer in sister dx at 33; breast cancer in mother dx 47, lang cancer mother's father
POL	PCR	BRCA1	NM_007294.3:c.5266dup	breast	45	ductal	Familial	2		breast cancer in sister dx at 43; brain cancer in mother died at 65; breast cancer in mother's sister
POL	PCR	BRCA1	NM_007294.3:c.5266dup	breast bilateral	49	ductal (papillary)	Hereditary	3		breast cancer in mother dx at 46 died at 49; breast cancer in mother's sister dx at 30 died 37; breast cancer in grandmother's brother's daughter dx at 43 died at 45
POL	PCR	BRCA1	NM_007294.3:c.5266dup	ovarian	32	serous	Hereditary			liver cancer in grandmother (father side) died at 60
POL	PCR	BRCA1	NM_007294.3:c.5266dup	ovarian	39	serous	Hereditary	1	1	breast cancer in sister dx at 41, ovarian cancer in mother dx at 35-40; colorectal cancer in father died at 73
POL	PCR	BRCA1	NM_007294.3:c.5266dup	ovarian	48	serous	Hereditary		3	ovarian cancer in sister dx at 31; ovarian cancer in mother dx 65 died 68; ovarian cancer in grandfather (mother side) dx at 59 died at 69; colorectal cancer in mother's brother dx at 62
POL	PCR	BRCA1	NM_007294.3:c.5266dup	ovarian bilateral	53	NA	Hereditary		2	ovarian cancer in mother dx at 45 died at 47; ovarain cancer in grandmother (mothers side), uterine cancer in sister
POL	PCR	BRCA1	NM_007294.3:c.5266dup	breast	30	medullary	Hereditary	3	1	breast cancer in 3 mother's sisters I dx at 36 died at 42; II dx at 51, III dx at 46; ovarian cancer in another mother's sister dx at 44; but mutation was detected in father!
POL	PCR	BRCA1	NM_007294.3:c.5266dup	breast and ovarian	53, 58	NA	Hereditary		1	lung cancer in mother died at 72; ovarian cancer in mother's sister dx at 58; melanoma in grandmother (maternal side); colorectal cancer in father's sister died at 60
POL	PCR	BRCA1	NM_007294.3:c.5266dup	breast bilateral	32, 36	medullary / ducta	Hereditary			no cancer
POL	PCR	BRCA1	NM_007294.3:c.5266dup	breast	39	ductal	Hereditary			uterine cancer in mother dx at 35 died at 37; lung cancer in father died at 65
POL	PCR	BRCA1	NM_007294.3:c.5266dup	breast	43	ductal	Hereditary		1	ovarian cancer in mother dx at 53 died at 53; lung cancer in grandfather (maternal side)
POL	PCR	BRCA1	NM_007294.3:c.5266dup	breast bilateral	30, 40	lobular / ductal	Hereditary	1		breast cancer in grandmother (maternal side) died at 50; uterine cancer in father's sister dx at 63
POL	PCR	BRCA1	NM_007294.3:c.5266dup	breast and ovarian	36, 51	solidum / serous	Hereditary			melanoma in mother died at 64; uterine cancer in mother's sister; colorectal cancer in second mother's sister, uterine cancer in father's sister died at 55; skin cancer in father's brother
POL	PCR	BRCA1	NM_007294.3:c.5266dup	breast bilateral	41, 51	medullary	Hereditary	2		breast cancer in mother; breast cancer in brother's daughter; melanoma in brother, stomach cancer in father's brother; larynx cancer in mother's brother; lang cancer in second mother's brother; stomach cancer in third mother's brother
POL	PCR	BRCA1	NM_007294.3:c.5266dup	ovarian	34	serous	Hereditary			no cancer
POL	PCR	BRCA1	NM_007294.3:c.5266dup	ovarian bilateral	44	serous	Hereditary		1	ovarian cancer in sister dx at 52
POL	PCR	BRCA1	NM_007294.3:c.5266dup	ovarian bilateral	49	serous	Hereditary	1	1	breast and ovarian cancer in grandmother (paternal side) died at 52; pancreas cancer in father's brother died at 52
POL	PCR	BRCA1	NM_007294.3:c.5266dup	breast, ovarian	34, 46	ductal (papillary)	Hereditary	2		breast cancer in sister dx at 36; breast cancer in mother dx at 45 died at 69; melanoma in mother's brother dx at 67 and maternal grandmother dx at 89; lung cancer in paternal grandfather; melanoma in paternal grandmother
POL	PCR	BRCA1	NM_007294.3:c.5266dup	ovarian	49	NA	Hereditary		1	ovarian cancer in mother
POL	PCR	BRCA1	NM_007294.3:c.5266dup	ovarian bilateral	46	mucinous	Hereditary		1	ovarian cancer in mother died at 54; uterine cancer in mother's sister, prostate cancer in mother's brother, lang cancer in mother's brother
POL	PCR	BRCA1	NM_007294.3:c.68_69del	breast	52	ductal	Sporadic			no cancer
POL	NGS	BRCA2	NM_000059.3:c.3075_3076delinsT	ovarian bilateral	42	adenocarcinoma	Hereditary	1		breast cancer in mother's sister dx at 60, stomach cancer in father dx at 60
POL	NGS	BRCA2	NM_000059.3:c.3075_3076delinsT	ovarian	52	endometroid	Familial		1	ovarian cancer in sister dx at 51, lung cancer in mother dx at 75, liver cancer in grandmother (from mother side) dx at 81
POL	NGS	BRCA2	NM_000059.3:c.3075_3076delinsT	ovarian	58	adenocarcinoma	Familial		1	ovarian cancer in sister dx at 52, lung cancer in mother 74, bones cancer mother's sister dx 60
POL	NGS	BRCA2	NM_000059.3:c.5205_5208del	breast	59	NA	Sporadic			colorectal cancer in father's sister
POL	NGS	BRCA2	NM_000059.3:c.5857G>T	breast bilateral	34	lobular	Hereditary	1		breast cancer in sister dx at 39; liver cancer in father's sister and mother's brother
POL	NGS	BRCA2	NM_000059.3:c.6315_6318del	breast	59	ductal in situ	Sporadic			no cancer
POL	NGS	BRCA2	NM_000059.3:c.8623G>T	breast bilateral	28	ductal	Hereditary	2		breast cancer in sister dx at 52; breast cancer in father's sister died at 40
POL	NGS	BRCA2	NM_000059.3:c.9253dup	breast, ovarian, colorec	51, 53, 55	ductal/ serous/ a	Hereditary			uterine cancer in mother died at 46; leukemia in father died at 36
UKR	NGS	BRCA1	NM_007294.3:c.1510del	breast	63	ductal	Hereditary	1		breast cancer in mother
UKR	NGS	BRCA1	NM_007294.3:c.181T>G	breast	34	NA	Hereditary	1		breast cancer in mother
UKR	NGS	BRCA1	NM_007294.3:c.181T>G	breast	42	NA	Hereditary	2		breast cancer in mother and aunt
UKR	NGS	BRCA1	NM_007294.3:c.181T>G	breast	41	lobular	Hereditary	1		breast cancer in mother
UKR	NGS	BRCA1	NM_007294.3:c.2217dup	breast	35	ductal	Hereditary	3		breast cancer in mother, grandmother and aunt, uterine cancer in aunt
UKR	NGS	BRCA1	NM_007294.3:c.2291_2292del	breast	45	ductal	Hereditary		1	ovarian cancer in mother
UKR	NGS	BRCA1	NM_007294.3:c.4357+1G>C	breast	46	lobular	Hereditary		2	ovarian cancer in mother and grandmother
UKR	NGS	BRCA1	NM_007294.3:c.5030_5033del	breast bilateral	40	ductal	Familial	1		breast cancer in sister
UKR	NGS	BRCA1	NM_007294.3:c.5030_5033del	breast	60	low differentiate	Hereditary		1	ovarian cancer in mother
UKR	NGS	BRCA1	NM_007294.3:c.5030_5033del	ovarian cancer	61	NA	Hereditary		1	ovarian cancer in mother
UKR	NGS	BRCA1	NM_007294.3:c.5177_5180del	breast	43	ductal	Hereditary	2		breast cancer in mother and sister
UKR	NGS	BRCA1	NM_007294.3:c.5177_5180del	breast	37	ductal	Hereditary	2		breast cancer in mother and sister
UKR	NGS	BRCA1	NM_007294.3:c.5266dup	breast	29	lobular	Hereditary	3		breast cancer in mother, grandmother and aunt
UKR	NGS	BRCA1	NM_007294.3:c.5266dup	breast	51	NA	Hereditary	1		breast cancer in twin sister
UKR	NGS	BRCA1	NM_007294.3:c.5266dup	breast	39	medullary	Familial	1		braest cancer in cousin (woman)
UKR	NGS	BRCA1	NM_007294.3:c.5266dup	breast	48	ductal	Familial		1	ovarian cancer in grandmother
UKR	NGS	BRCA1	NM_007294.3:c.5266dup	breast	34	ductal	Hereditary	2		breast cancer in mother aunt
UKR	NGS	BRCA1	NM_007294.3:c.5266dup	breast	37	ductal	Hereditary	1		breast cancer in mother
UKR	NGS	BRCA1	NM_007294.3:c.5266dup	breast bilateral	59	ductal	Hereditary	2		breast cancer in mother and cousin (man), uterine cancer in aunt
UKR	NGS	BRCA1	NM_007294.3:c.5266dup	breast	42	ductal	Hereditary	4		breast cancer in mother, grandmother, daughter and aunt
UKR	NGS	BRCA1	NM_007294.3:c.5266dup	breast	32	low differentiate	Hereditary	4		breast cancer in mother, grandmother, grand-grandmother and aunt
UKR	NGS	BRCA1	NM_007294.3:c.5266dup	breast	55	ductal	Hereditary	3	1	breast cancer in mother, first cousin (woman) and niece; ovarian cancer in grandmother
UKR	NGS	BRCA1	NM_007294.3:c.5266dup	breast	50	ductal	Hereditary	4		breast cancer in mother, grandmother, aunt, first cousin (woman)
UKR	NGS	BRCA1	NM_007294.3:c.68_69del	ovarian, breast	54, 69	ductal	Hereditary		5	ovarian cancer in mother, grandmother, aunt, sister and first cousin (woman)
UKR	NGS	BRCA1	NM_007294.3:c.68_69del	ovarian	48	NA	Hereditary	1		breast cancer in sister
UKR	NGS	BRCA1	NM_007294.3:c.844_850dup	breast	41	ductal	Hereditary	4		breast cancer in mother, sister and two aunts
UKR	NGS	BRCA2	NM_000059.3:c.10095delinsGAAT	breast	57	ductal	Familial	1		breast cancer in grandmother
UKR	NGS	BRCA2	NM_000059.3:c.2945del	breast bilateral	60	NA	Hereditary	1		breast cancer in sister
UKR	NGS	BRCA2	NM_000059.3:c.475+1G>T	breast	29	ductal	Hereditary	2		breast cancer in mother and aunt
UKR	NGS	BRCA2	NM_000059.3:c.475+1G>T	breast	44	lobular	Familial		1	breast cancer in sister
UKR	NGS	BRCA2	NM_000059.3:c.475+1G>T	breast bilateral	59	ductal	Hereditary			uterine cancer mother
UKR	NGS	BRCA2	NM_000059.3:c.6405_6409del	breast	41	ductal	Hereditary	2		breast cancer in grandmother and twin sister
UKR	NGS	BRCA2	NM_000059.3:c.6408_6414del	breast	33	ductal	Hereditary	4		breast cancer in mother, grandmother and two aunts
UKR	NGS	BRCA2	NM_000059.3:c.7069_7070del	breast	41	ductal	Hereditary		1	ovarian cancer in mother
UKR	NGS	BRCA2	NM_000059.3:c.7721G>A	breast	45	ductal	Familial	2		breast cancer in two aunts
UKR	NGS	BRCA2	NM_000059.3:c.9097dup	breast	NA	NA	NA	NA	NA	NA
